# Informing investment in health workforce in Bangladesh: a health labour market analysis

**DOI:** 10.1186/s12960-022-00769-2

**Published:** 2022-10-12

**Authors:** Md Nuruzzaman, Tomas Zapata, Michelle McIsaac, Sangay Wangmo, Md Joynul Islam, Md Almamun, Sabina Alam, Md Humayun Kabir Talukder, Gilles Dussault

**Affiliations:** 1 WHO Bangladesh, Dhaka, Bangladesh; 2 WHO South-East Asia Regional Office (Former HRH Advisor), New Delhi, India; 3 WHO Health Workforce Department, Geneva, Switzerland; 4 Health Services Division, Ministry of Health and Family Welfare, Dhaka, Bangladesh; 5 Centre for Medical Education, Directorate General of Medical Education, Dhaka, Bangladesh; 6 Global Health and Tropical Medicine, Instituto de Higiene E Medicina Tropical, Universidade NOVA de Lisboa, Lisbon, Portugal

**Keywords:** Health labour market analysis, Health workforce, Supply, need and demand, Investment, Bangladesh, WHO

## Abstract

**Background:**

As the 2016 Global Strategy on Human Resources for Health: Workforce 2030 (GSHRH) outlines, health systems can only function with health workforce (HWF). Bangladesh is committed to achieving universal health coverage (UHC) hence a comprehensive understanding of the existing HWF was deemed necessary informing policy and funding decisions to the health system.

**Methods:**

The health labour market analysis (HLMA) framework for UHC cited in the GSHRH was adopted to analyse the supply, need and demand of all health workers in Bangladesh. Government’s information systems provided data to document the public sector HWF. A national-level assessment (2019) based on a country representative sample of 133 geographical units, served to estimate the composition and distribution of the private sector HWF. Descriptive statistics served to characterize the formal and informal HWF.

**Results:**

The density of doctors, nurses and midwives in Bangladesh was only 9.9 per 10 000 population, well below the indicative sustainable development goals index threshold of 44.5 outlined in the GSHRH. Considering all HWFs in Bangladesh, the estimated total density was 49 per 10 000 population. However, one-third of all HWFs did not hold recognized roles and their competencies were unknown, taking only qualified and recognized HWFs into account results in an estimated density 33.2. With an estimate 75 nurses per 100 doctors in Bangladesh, the second area, where policy attention appears to be warranted is on the competencies and skill-mix. Thirdly, an estimated 82% of all HWFs work in the private sector necessitates adequate oversight for patient safety. Finally, a high proportion of unfilled positions in the public sector, especially in rural areas where 67% of the population lives, account only 11% of doctors and nurses.

**Conclusion:**

Bangladesh is making progress on many of the milestones of the GSHRH, notably, the establishment of the HWF unit and reporting through the national health workforce accounts. However, particular investment on strengthening the intersectoral HWF coordination across sectors; regulation for assurance of patient safety and adequate oversight of the private sector; establishing accreditation mechanisms for training institutions; and halving inequalities in access to a qualified HWF are important towards advancing UHC in Bangladesh.

## Introduction

Bangladesh aspires to achieve the Sustainable Development Goals (SDGs) including Universal Health Coverage (UHC) by 2030 [1]. This calls for ensuring access to quality healthcare for all at an affordable cost so that no one faces financial hardship because of health problems. However, the country’s estimated density of 9.9 doctors, nurses and midwives per 10 000 population in 2019 is far below the global median of 48.6 [2, 3] and the indicative threshold of 44.5 per 10 000 population for attainment of sustainable development goals (SDGs) outlined in the WHO *Global Strategy on Human Resources for Health: Workforce 2030* [4]. The global workforce strategy was adopted by the Member States in the Sixty-Ninth World Health Assembly with the vision of accelerating progress towards UHC and SDGs by ensuring universal access to health workers [5].

The Government of Bangladesh’s (GOB) *Bangladesh Health Workforce Strategy-2015* urges the determination of service level-wise (i.e. primary, secondary and tertiary levels) health workforce needs and project demands to 2030 [6]. The *Strategy* also calls for a health workforce production and development plan to help identify supply-side bottlenecks and ensure the availability of a competent health workforce.

Health services system in Bangladesh is pluralistic in nature, where multiple actors, e.g. public and private sectors, and autonomous bodies involved in healthcare delivery [7]. However, the government has the constitutional obligation to adopt all appropriate policies, plans and programmes to ensure basic health care for all [8]. In line with the *Global Strategy*, the Ministry of Health and Family Welfare (MOHFW), with WHO’s technical assistance conducted a comprehensive analysis of the health labour market in order to align the public policy agenda with strategic intelligence on the entirety of the national health workforce. A health labour market analysis (HLMA) approach can provide a better understanding of the factors that drive health workforce shortages and surpluses, imbalances in skill-mix and geographical distribution and suboptimal performances. It also helps to raise awareness among the policy-makers and relevant stakeholders of how and why labour market changes and to adopt appropriate remedial actions [9].

This paper outlines the findings of this HLMA. It highlights progress already made and left to be made by Bangladesh on several *Global Health Workforce Strategy* milestones. One area that was particularly notable given the results of the HLMA was that of ensuring adequate oversight of the private sector and increased investment in the public sector and its health workforce, especially in rural areas.

## Methods

The HLMA used the framework outlined in the *Global Strategy* [4]. Understanding the dynamics of the health labour market involves an analysis of two distinct but closely related economic forces: the demand for health workers and their supply, whose dynamic relationship is shaped by a country’s institutions and regulations. The demand for health workers in the market can be defined as the willingness and ability of the government, private sector and/or donors to pay to have health workers placed in clinics, hospitals or other parts of the health system. The supply refers to how many health workers are available at any given moment and how many hours they are willing to work.

For the public sector workforce, the main source of data was the MOHFW. Public workforce data were available on health education institutions, including admissions and graduates, and on the number and type of workers employed and of positions available. Bangladesh has made good progress during 2015–2018 on health workforce registries to track health workforce stock, distribution, flows, demand, supply, capacity and remuneration in the public sector [10, 11]. However, the public sector employed only a small portion of the health workers in Bangladesh [7, 8].

Therefore, gathering information on the private sector was a priority for a better understanding of the entirety of the national health labour market. The MOHFW and WHO with the support of the Bureau of Statistics conducted a national survey in 2019 based on a sample of 133 geographical units representative of the whole country that estimated the composition and characteristics of the health workforce in the public, private and informal sectors [12]. The MOHFW established a technical working group to supervise the 2019 survey.

A rapid search of the published literature and interviews of key-informants provided additional information. Interviewees included the State Minister of Health and Family Welfare, Health Secretary-Medical Education and Family Welfare Division, Additional Secretary-Administration of the Health Services Division, Director-Medical Education and Health Manpower Development of the Directorate General of Health Services, Director General-Public Administration, Vice-chair BRAC, Registrars of the Bangladesh Medical and Dental Council, and Nursing and Midwifery Council [12].

A MOHFW Advisory Group (Steering Committee) oversaw the HLMA process. It included representatives from different departments under MOHFW, Expatriate’s Welfare and Overseas Employment, Public Administration, Foreign Commonwealth Development Office of the UK, Global Affairs Canada, USAID and WHO Bangladesh [12].

The HLMA used descriptive statistics to portray a complete picture of the health workforce in Bangladesh, in terms of headcounts. In many countries, this would be an important limitation and full-time equivalents would be preferred. In Bangladesh, public sector workers are all full-time and we assumed, based on the 2019 survey, that it was generally the case in the private sector.

## Results

This section presents the findings of the HLMA in four parts: (1) an overview of the “production” of health workers (education institutions and foreign-trained); (2) a description of supply and demand in the public sector, followed by the (3) supply in the private sector.

### The education pipeline and foreign-trained workers (production)

The production from education institutions and inflows of foreign-trained workers determine the various dimensions of the health workforce: its size and composition (availability), its distribution by levels of care, type of facility and geographical zones (accessibility) and the competencies they acquire, their alignment with population needs and how workers apply them (acceptability, quality coverage). Data to 2016 for health worker education institutions are from a mapping study conducted by the MOHFW and WHO (2018) and more recent ones are from the MOHFW statistics [13].

#### Institutions

The MOHFW approves the establishment of health professional education institutions [13]. Respective professional councils (e.g. Bangladesh Medical and Dental Council, and Bangladesh Nursing and Midwifery Council), and boards (e.g. Bangladesh Homeopathy Board) provide accreditation to these institutions. There has been a major increase of the number of health worker education and training institutions after 2010, namely in the area of nursing and midwifery (Table 1).

**Table 1:** Total number of health worker education and training institutions (public, including armed forces, and private), 2010, 2016 and 2020

Type of institution	Degree and length	2010	2016	2020
Medical colleges	Bachelor, 5 years	62	105	113
Dental colleges	Bachelor, 5 years,	17	35	35
Nursing colleges	Bachelor, 4 years	30	64	174
Nursing institutes (nursing and midwifery)	Diploma, 3 years	57	157	223
Sub-Assistant Community Medical Officer (SACMO)Training Schools	Diploma, 3 years	47	208	209
Institutes of health technology (offering Diploma in Medical Technology-pharmacy)	Diploma, 3 years	61 (35)	105 (51)	110 (54)
No. of universities offering bachelor’s degree in pharmacy	Bachelor, 4–5 years	32	36	41
Total		306	710	905

This expansion translated in increased numbers of seats and of graduates. In public medical colleges, the number of seats available went from 2920 before 2010 to 4475 in 2020, a 53% increase; in private colleges, the figures are 3669 before 2010 and 6597 in 2020 (+80%). Public dental colleges offered 216 seats before 2010 and 532 (+146%) in 2020 and private ones 740 before 2010 and 1405 (+90%) in 2020.

In 2020, private education institutions offered more seats in total: 59% of seats for MBBS studies, 89% for BSc Nursing and 94% for SACMO (Table 2). Between 2008 and 2018 (last year available), private colleges had produced 52% of the 55 442 medical graduates in the country. This proportion is likely to keep increasing as students from colleges created since 2015 are starting to graduate.

**Table 2:** Number of public and private sector health worker education institutions and seats available, 2020

Institutions/sector	Medical colleges (# of seats)	Dental colleges (# of seats)	Nursing colleges/BSC (# of seats)	Nursing Institutes/diploma (# of seats)	Midwifery institutes (# of seats)	Sub-Assistant Community Medical OfficerTraining Schools (# of seats)	Technology institutes (# of seats)
Public	38 (34%), 4475 (41%)	9 (26%), 532 (27%)	32 (18%), 1935 (11%)	44 (20%), 3380 (28%)	41 (37%), 1050 (28%)	9 (4%), 816 (6%)	13 (12%), 2526 (20%)
Private	75 (66%), 6594 (59%)	26 (74%), 1405 (73%)	142 (82%), 15 145 (89%)	179 (80%), 8705 (72%)	71 (63%), 2690 (72%)	200 (96%), 13 185 (94%)	97 (88%), 8865 (80%)
Total	113 (11 069)	35 (1937)	174 (17 080)	223 (12 085)	112 (3740)	209 (14 001)	110 (11 391)

Source: Health Labour Market Database 2021, Human Resource Branch, Health Services Division, Ministry of Health and Family Welfare, Bangladesh

There is no alignment between the geographical distribution by Region of education institutions and of seats available with the distribution of the population (Table 3). There is a concentration of seats in Dhaka for all occupational categories; the Region has 18% of the population of the country, but 65% of seats in dental colleges, 52% in technology institutes, 48% in medical colleges, 46% in nursing colleges, 41% in SACMO schools, and 30.9% in nursing institutes. Only Mymensingh has a number of seats corresponding to its proportion of its total population. Private SACMO schools offer 93.8% of total seats, nursing colleges 88.6%, technology institutes 78%, dental colleges 72.5%, nursing institutes 72%, and medical colleges 59.3% (Table 3).

**Table 3:** Number of public and private health worker education institutions, number and percentage of seats, by Division/Region

Division (% of population)	No. of medical colleges, seats and % of total seats			No. of nursing colleges, seats and % of total seats			No. of nursing institutes, seats and % of total seats			No. of midwifery institutes, seats and % of total seats			No. of dental colleges, seats and % of total seats			No. of medical assistant training schools, seats and % of total seats		
	Total	Public	Private	Total	Public	Private	Total	Public	Private	Total	Public	Private	Total	Public	Private	Total	Public	Private
Rajshahi (22.12%)	12 (1110, 10.38%)	4 (545, 12,5%)	8 (565, 8.9%)	28 (2585, 15.13%)	5 (175, 9%)	23 (2410, 15.9%)	42 (2215, 18.33%)	6 (510, 15.1%)	36 (1705, 19,6%)	16 (465, 12.4%)	7 (175, 16.7%)	9 (290, 10.8%)	4 (149, 7.69%)	1 (59, 11,1%)	3 (90, 6,4%)	45 (2932, 20.94%)	1 (102, 12,5%)	44 (2830, 1,5%)
Dhaka (17.82%)	55 (5142, 48.6%)	10 (1257 28.9%)	45 (3885, 61.2%)	77 (7785, 45.58%)	10 (560 28.9%)	67 (7225 47.7%)	69 (3745, 30.99%)	10 (810 24%)	59 (2935 33,7%)	40 (1425, 38.1%)	10 (275, 26.2%)	30 (1150, 42.8%)	21 (1260, 65%)	3 (205, 38,5%)	18 (1055, 75,1%)	78 (5714, 41%)	2 (204, 25%)	76 (5510, 41,8%)
Chattogram (14.06%)	17 (1391, 12.54%)	6 (651, 15%)	11 (740, 11.7%)	20 (1345, 7.87%)	5 (350, 18.1%)	15 (995, 6.6%)	16 (1110, 9.18%)	5 (465, 13.8%)	11 (645, 7,4%)	12 (345, 9.2%)	7 (175, 16.7%)	5 (170, 6.3%)	2 (125, 6.45%)	1 (60, 11,3%)	1 (65, 4,6%)	13 (819, 5.9%)	2 (154, 18,9%)	11 (665, 5%)
Rangpur (13.92%)	7 (800, 7.48%)	3 (460, 10.6%)	4 (340, 5.4%)	14 (1460, 8.55%)	4 (425, 22%)	10 (1035, 6.8%)	35 (1855, 5.54%)	5 (305, 9%)	30 (1550, 17,8%)	13 (430, 11.5%)	4 (100, 9.5%)	9 (330, 12.3%)	2 (152, 7.84%)	1 (52, 9,8%)	1 (100, 7,1%)	22 (1410, 10%)	0	22 (1410, 0,7%)
Sylhet (11.21%)	6 (743, 6.95%)	3 (331, 7.6%)	3 (412, 6.5%)	10 (1300, 7.61%)	1 (125, 6.5%)	9 (1175, 7.8%)	10 (475, 3.93%)	2 (150, 4.4%)	8 (325, 3,7%)	8 (285, 7.6%)	3 (75, 7.1%)	5 (210, 7.8%)	3 (117, 6.04%)	1 (52, 9,8%)	2 (65, 4,6%)	7 (500, 3,5%)	0	7 (500, 3,8%)
Khulna (9.71%)	10 (755, 7.06%)	6 (480, 11%)	4 (272, 4.3%)	8 (530, 3.1%)	4 (50, 2.6%)	4 (480, 3.2%)	26 (1400, 11.58%)	8 (635, 18.8%)	18 (765, 8,8%)	8 (250, 6.7%)	5 (125, 11.9%)	3 (125, 4.6%)	0	0	0	25 (1581, 11.29%)	4 (356, 43,6%)	21 (1225, 9,3%)
Barishal (6.83%)	2 (331, 3.09%)	2 (281, 6.5%)	0	10 (1125, 6.59%)	2 (125, 6.5%)	8 (1000, 6.6%)	12 (670, 5.54%)	5 (355, 10.5%)	7 (315, 3,6%)	8 (275, 7.4%)	4 (100, 9.5%)	4 (175, 6.5%)	1 (52, 2.68%)	1 (52, 9,8%)	0	6 (375, 2.67%)	0	7 (375, 2.8%)
Mymensingh (4.32%)	4 (475, 4.44%)	3 (345, 7.9%)	1 (130, 2%)	7 (950, 5.56%)	1 (125, 6.5%)	6 (825, 5.4%)	13 (615, 5.09%)	3 (150, 4.4%)	10 (465, 5,3%)	7 (265, 7.1%)	1 (25, 2.4%)	6 (240, 8.9%)	2 (82, 4.23%)	1 (52, 9,8%)	1 (30, 2,1%)	12 (670, 4,7%)	0	12 (670, 5,1%)
Bangladesh	113 (10 694)	38 (4350)	75 (6344)	174 (17 080)	32 (1935)	142 (15 145)	223 (12 085)	44 (3380)	179 (8705)	112 (3740)	41 (1050)	71 (2690)	35 (1937)	9 (532)	26 (1405)	209 (14,001)	9 (816)	200 (13,185)

Source: Health Labour Market Database 2021, Human Resource Branch, Health Services Division, Ministry of Health and Family Welfare, Bangladesh

#### Students

The demand (pool of candidates seeking admission) for health worker education is very large and the further expansion of the “production” would be possible; for example, there are 9 applicants per seat for admission to MBBS studies [13]. Such expansion is possible only if the capacity of education institutions permits (infrastructures equipment clinical training settings, educators and trainers), which is a major challenge.

The “production” of medical doctors and other professionals, namely nurses and midwives, has increased nationally in the last decade due to the growth especially the private sector (Table 2). In public institutions, in 2020, there were more seats for medical than for nursing studies, (4475 vs 5315, of which 1935 for BSC nursing). When public and private seats for BSc nursing are added, their number is still inferior to that of total medical seats, 17 080 vs 11 072 (Table 2).

Between 2008 and 2018, 55% of all medical graduates were women (52% in 2008 and 59% in 2018). During that period, an equal number of men and women graduated from public colleges; the proportion of women graduates was 59% in private colleges (Table 3). The trend is similar for dentists. Between 2007 and 2016, 66% of BDS graduates were women; the proportion from public dental colleges was 56% and of 79% from private ones. There have been 3516 Diploma in Medical Technology (DMT) graduates in pharmacy, 1048 in public IHT and 2468 in private ones; 36% were women. Admissions in 2016 were 268 in public IHT, and 72 in private ones. Data for 2020 are not available.

### The supply and demand of health workers in the public sector

Demand for health workers in the public sector corresponds to the number of sanctioned posts offered by public employers. Data are available only for the MOHFW, which employs most public sector health workers; it shows important gaps between supply and demand for most categories of workers.

Table 4 shows unmet demand of more than one-third of available positions for doctors, dentists, midwives and medical technologists. The gap is lower for nursing staff and the Directorate General of Nursing and Midwifery is the Directorate with less unfilled posts. In total, clinical staff represent 61.3% of all unfilled posts; domiciliary staff and alternative medicine account for the remainder. Among medical technologists, the highest percentage of unfilled posts is for physiotherapists (77%), followed by laboratory technologists (44%) and radiotherapists (43%) and pharmacists (42%) [14].

**Table 4:** Health workers employed by the Ministry of Health and Family Welfare (DGHS and DGNM), by occupational category (2021)

Health worker category	Sanctioned posts	Filled posts	% Vacant posts
Doctors	40 162	26 619	33.7
Dentists	1361	829	39.1
Nurses	40 015	35 828	10.5
Sub-Assistant Community Medical Officers (SACMOs)	5397	3.661	32.5
Midwives	2996	1145	61.7
Medical Technologists (total eight sub-categories)	6406	3892	39.2
Domiciliary staff	75 009	59 183	21.1
Alternative medicine	1906	1053	44.7
Pharmacists (Category B)	2982	1744	41.5
Total	176 234	133 210	24.0

Source: DGHS, DGNM and DGFP under the MOHFW (2021)

#### Composition of health workforce in the public sector

In 2019, the MOHFW employed 151 532 individuals: 74 985 in the Directorate General of Health Services (DGHS), 41 282 in the Directorate General of Family Planning (DGFP) and 35 265 in the Directorate General of Nursing and Midwifery (DGNM) [15]. DGHS, DGFP and DGNM collectively employed more than 95% of the total workforce under the MOHFW [15]. In total, 58.6% were women; this proportion was 36.4% for doctors and 90% for nurses. The number of nurses, including BSC and diploma, per doctor is 1.35; it is 1.04 if Sub-Assistant Community Medical Officers (SACMOs) are added to doctors (Table 5).

**Table 5:** Health workers employed by the Ministry of Health and Family Welfare (in DGHS, DGFP and DGNM), by sex and occupational category (2021) [12, 14, 16, 17]

Health worker category	Total	Male	Female
Doctors	26 619	16 919 (64%)	9700 (36%)
Dentists	829	467 (56%)	362 (44%)
Nurses (BSc and Diploma)	35 828	3582 (10%)	32 246 (90%)
Sub-Assistant Community Medical Officer (SACMO)	7927	5459 (69%)	2468 (31%)
Midwifery	1145	0 (0%)	1145 (100%)
Medical technologists	6248	5276 (84%)	972 (16%)
Domiciliary staff^*^	57 451	22 071 (38%)	35 380 (62%)
Alternative medicine	1053	705 (67%)	348 (33%)
Pharmacists-Category B**	1744	1411 (81%)	333 (19%)
Total HWF	138 844	55 890 (40%)	82 954 (60%)

^*^Domiciliary staff refers to includes health inspector, assistant health inspector and health assistant

^**^Pharmacist-Category B refers to the medical technologists with the 3 years Diploma in Pharmacy

The major expansion of education institutions in the last decade contributed to building a young health workforce; 57.3% of doctors, 61% of dentists, 70% of nurses and 78% of midwives are less than 40 years old. Notably, only 9.2% of the doctors and 0.02% of the nurses are over 59 years old (Table 6).

**Table 6:** Age distribution of health workers employed by the Ministry of Health and Family Welfare by occupational category (%), 2021

Category	< 25–29 (%)	30–39 (%)	40–49 (%)	50–59 (%)	> 59 (%)	Total (*N*)
Doctors	15.50	41.81	21.11	12.39	9.20	26 695
Dentists	18.98	42.09	21.17	12.17	5.60	822
Nurses	30.85	30.60	22.95	15.58	0.02	30 375
Midwifes	74.12	8.98	13.37	3.42	0.11	935
Medical Technologists	4.60	32.40	31.22	31.73	0.05	3892
Sub-Assistant Community Medical Officer	13.90	51.46	17.24	16.74	0.66	3661
Medical Technologist-Pharmacy	8.2	41.51	27.47	21.9	0.92	1744

#### Availability and accessibility

Nationally, the total density of health workers employed by the MOHFW is low at approximately 8 per 10 000 population. Data on densities by occupational category show that nurses (BSC and diploma) have the highest at 2.13, followed by doctors at 1.72, composed of 1.45 for generalists and 0.27 for specialists (Table 5). Data by administrative division indicate a concentration of doctors in the Dhaka Division, whereas the other categories are more evenly distributed. With the exception of SACMOs who all work in rural and hard-to-reach zones, between 50 and 75% of the other occupational categories work in urban zones, where only 38% of the population live. Most importantly, 75.3% of the doctors and 75% of the nurses work in urban areas. Between 50 and 75% of all occupational categories work in tertiary services, except for SACMOs who all work at primary care level. It is therefore important to address the recurring factors that influence attraction, development, recruitment, and retention of health workers in rural areas [18].

### Availability, composition and accessibility of the non-government (private) health workforce

The 2019 survey estimates a density of 40.4 per 10 000 population of non-government workers, of which 15.2 corresponds to non-qualified and non-recognized workers [12]. The informal sector includes the following providers: untrained Physiotherapist (with no academic degree), Drug/Medicine Seller, Unani care provider (with no formal education/training), Ayurveda care provider (with no formal education/training), Kaviraj, Traditional Birth Attendant, Palli Chikitshok (Village doctor)—Untrained Totka, Dental Technician (without formal education or training), Lab Technician (without formal education or training). The supply of non-qualified and non-recognized health worker’s density per 10 000 population is 15.21, representing 31% of the total supply of health workers. In a 2008 health workforce study conducted by BRAC, informal providers were about 66% of the total [7]. They represent 37% of workers in the private sector. Four divisions have a density superior to the national one, more than twice in the case of Dhaka. The most numerous informal workers are drug sellers, village doctors and traditional birth attendants in that order. About 31% are women and about 18% are less than 35 years old, 38% are between 36 and 55 and 44% are above 56. About 79% did not reach the diploma level. On average, they have been active for 15.8 years. About 84% are self-employed, serving 26 patients per day on average, and earning 8875 takas per month (USD 103). No data is available on demand for recognized health workers in the private sector [12] (Table 7).

**Table 7:** Distribution of informal (non-qualified and non-recognized) health workers by Division, estimated number and density [12]

Division (% of total population in Bangladesh)	Number of informal workers (% of total)	Density per 10 000 pop	Density per 10 000 pop. urban	Density per 10 000 pop. rural
Barishal (6.83%)	17 813 (7.3%)	16.28	48.03	15.1
Chattogram (14.06%)	21 914 (9%)	9.73	15.87	6.73
Dhaka (17.82%)	106 408 (43.6%)	37.27	52.47	9.46
Khulna (9.71%)	28,594 (11,7%)	18.38	17.76	18.74
Mymensingh (4.32%)	14 180 (5.8%)	20.46	11.58	26.6
Rajshahi (22.12%)	25 547 (10.5%)	7.21	11.48	6.09
Rangpur (13.92%)	20 157 (8.3%)	9.04	10.33	7.26
Sylhet (11.21%)	9141 (3.8%)	5.21	3.99	5.51
Total	243 754	15.21	24.64	10.17

## Discussion

### Progress on health workforce registries

Although data from the public sector are available and reliable, it covers only an estimated one-fifth of the national health labour market. There is a clear need to ensure reliable and timely collection of data on all health workers in both the government and non-government sectors. WHO’s National Health Workforce Accounts (NHWA) ask Member States to establish a strong mechanism ensuring timely and reliable data for evidence-based decision making [19]. It calls for strengthening of the capacity of professional councils, as data collectors, and of analysts to make this database a powerful tool for planning purposes. This is crucial, as the quality of estimates of future needs cannot be better than the quality of data and information available. The vision for a Digital Bangladesh by 2021 offers an opportunity to develop a robust and inter-operable HRIS in the MOHFW and its departments [20].

### Regulatory mechanisms to promote patient safety and adequate oversight of the private sector

The regulations safeguarding professional standards and quality and protection of people’s healthcare rights exist, but there is no systematic data collection on their implementation. The 2019 survey showed that more than 43% of medical doctors’ respondents were not able to show their license or registration certificate, which is against the rules of the Bangladesh Medical and Dental Council [21]. Areas in need of stronger regulation and oversight include private sector practice and education programmes, dual practice by government workers, mainly doctors, and practice by unqualified and unrecognized workers. The need for strengthening existing regulatory mechanism is also recommended in other South-East Asian countries, such as India, Nepal and Sri Lanka [22, 23].

Nearly one-third of the active health workforce in Bangladesh is unqualified and informal, a proportion that has gradually diminished as the number of qualified workers increased. Strategies should be developed to track and monitor of these workers and bring them under a regulatory and capacity development framework that mitigates potential risks associated to the utilization of their services. In the mid-term, the objective should be ensuring universal access to qualified workers (for example ensuring access to qualified pharmacists for all would reduce the utilization of drug sellers).

There is a need to assess the capacity of the six professional councils and boards in setting standards and managing registration and licensing. For example, the State Medical Faculty of Bangladesh, who registers medical technologists and medical assistants work on the basis of an outdated Act or law [13]. Accountability mechanisms showing how effective these councils and boards fulfil their mission is needed.

### Accreditation of health training institutions

The existing accreditation system is mainly driven by the MOHFW that plays a dual role in this regard. It formulates rules and policies and executes them through its own Directorates and agencies. WHO suggests the establishment of a strong accreditation system to ensure high-level professional standards [24].

### Development and monitoring of health workforce policies and plans

The MOHFW has a Human Resources for Health (HRH) Unit at its Secretariat. However, HRH functions remain scattered among different directorates and agencies, with limited institutional linkages, and coordination mechanisms for policy coherence and data sharing [25]. This is further hindered by the limited number of technical staff, which results no comprehensive planning in place [26]. The government has already developed multiple policies to improve the retention of health workers in rural areas. These include the allocation of 20% of seats in public medical schools to district students and of 5% of seats in private medical schools to poor students, clinical rotations in rural health facilities, rural service for public medical schools graduates, financial incentives and accommodation, but their effects seem limited. WHO has recently highlighted the critical importance that interventions to improve access to health workers in rural areas be interconnected, bundled and tailored to the local context [18]. An evaluation of the relevance, acceptability, feasibility, affordability, effectiveness and impact of these strategies, of their implementation and of their results would provide information to plan the workforce coverage more effectively [27].

### Unmet demand in the public sector

In the public sector, the high proportion of unfilled sanctioned positions indicates an important unmet demand for doctors, SACMOs, dentists, medical technologists and midwives; this proportion is 10.5% for nurses. All divisions experience this problem at more or less the same degree. DGHS MIS data (2020–2021) report that vacancy rates of medical doctors are about 80% at Union Sub-Center (primary care units) and range between 40 and 50% in Upazila Health Complexes (first referral health facility at primary care level) [16]. There is therefore unmet demand of all categories of qualified health workers in the public sector. Available data do not inform on the existence or absence of shortages in the private sector. Data going back to 1997 show that similar shortages have been a constant feature in the DGHS [28].

### Intersectoral health workforce agenda

The Government of Bangladesh has a constitutional obligation to ensure basic medical care for all [29]. In order to meet its goal of universal health coverage, the contribution of the public sector workforce to health care is key. This study indicates that it comprises only 18% of the total workforce contributing to health care delivery. This calls for the gradual increase of the budget allocation to healthcare and the creation of new jobs for qualified health workers [12, 30].

## Conclusion

Bangladesh has made great progress on many of the monitoring and accountability milestones of the Global Strategy. Examples of important efforts are the establishment in 2016 of a HRH Unit with responsibility for development and monitoring of policies and plans, and improved health workforce data collection and sharing through the National Health Workforce Accounts (NHWA). However, much of this progress took place in the public sector which comprises only an estimate 18% of the total health workforce in Bangladesh.

The HLMA highlighted several areas where policy attention might be necessary. Firstly, there is a clear need for mechanisms to regulate and collect data and information that cover all the health workers in Bangladesh. With a such a high proportion of health workers in the private sector, and with many in this private sector workforce falling outside of recognized occupational categories, it is of great importance to strengthen the regulatory oversight of this workforce. It is important to note that a gradual integration of the informal health workforce with the right competencies into formal care provision could align with the health and employment agendas of the SDGs.

Secondly, it remains important to increase the availability and accessibility of health workers in order to attain UHC, a particular focus on reducing the prevailing inequalities in accessing health workers that exist in Bangladesh is necessary. Health workers are inequitably distributed across levels of care and geographical areas. With health workers concentrated in urban areas and many public sector workers in tertiary care, consideration of how the supply of all health workers and the notably those that are part of the public sector are distributed is acutely needed. Access to health workers in rural areas with the lowest densities of recognized health workers is a policy issue closely tied to the primary health care agenda that needs full attention. The assessment of future health workers requirements is not only about supply in terms of numbers, but also about alignment to the global milestone on reducing inequality in access to a health worker, appropriate skill-mix, competencies, working conditions, productivity and quality.

Thirdly, on developing a better understanding of the competencies and skill-mix of the health workforce, the HLMA highlighted that there is an increase of the number of health worker education and training institutions, particularly in the private sector. The composition and skill-mix of graduates along with the quality of training and education needs to be addressed in parallel to such an expansion, to make a meaningful contribution to meeting the health needs of Bangladesh and alignment with the global milestone on accreditation mechanisms for health training institutions.

Since the HLMA initiative was first of this kind in Bangladesh, the Ministry of Health needs to strengthen the mechanism so that health workforce inflows and outflows to the labour market could be monitored on periodic basis, and that a balance in the supply and demand is ensured and shortage and surplus in the long run.

## Data Availability

The datasets used and/or analysed during the current study are available from the corresponding author on a reasonable request

## Abbreviations

BSc: Bachelor of Science
DGHS: Directorate General of Health Services
DGFP: Directorate General of Family Planning
DGNM: Directorate General of Nursing and Midwifery
DMT: Diploma in Medical Technology
GOB: Government of Bangladesh
HLMA: Health Labour Market Analysis
HRIS: Human Resources Information Systems
HRH: Human Resources for Health
HSD: Health Services Division
IHT: Institute of Health Technology
MATS: Medical Assistant Training School
NHWA: National Health Workforce Accounts
MOHFW: Ministry of Health and Family Welfare
SACMO: Sub-Assistant Community Medical Officer
UHC: Universal Health Coverage
WHO: World Health Organization
